# Supplementary material for: Neutrophil Extracellular Traps Drive Dacryolithiasis
Source: Cells. 2023 Jul 14;12(14):1857. doi: 10.3390/cells12141857 (PMC10377949; doi:10.3390/cells12141857)
Supplement: Supplementary file 1 [file cells-12-01857-s001.zip › cells-2478842-supplementary.pdf]

Table S1. Immune fluorescence of various antigens in MPC-Ds (all samples) [1]

Core	P94	P95	P96	P97	P98	P99	P102
citH3	0	0	0	0	0	1	0
MUC5B	0	0	0	0	0	1	0
MPO	0	0	0	0	0	1	1
Fetuin A	0	1	0	0	0	1	1
MUC5AC	0	1	1	0	1	1	0
CK-1	1	1	1	0	0	1	0
CK-10	1	1	1	1	1	1	0
Fibrinogen	1	2	1	0	0	1	1
AQP-9	1	0	2	0	0	2	2
NE	0	2	0	0	0	1	3
Hemoglobin	2	2	2	0	2	2	3
CK-2	0	0	3	0	3	2	2
CK-9	1	0	3	0	3	2	2
SNA	2	2	1	0	3	3	0
IgG	2	3	2	0	2	2	3
PAD4	2	3	2	0	2	3	3
GalNAc	3	3	3	1	3	3	1
Surface	**P94**	**P95**	**P96**	**P97**	**P98**	**P99**	**P102**
MUC5B	1	1	0	1	0	0	0
citH3	0	1	0	0	0	2	2
MPO	1	1	0	0	1	2	2
Hemoglobin	2	2	2	2	0	2	2
Fetuin A	0	1	0	0	0	0	3
MUC5AC	0	1	1	3	0	1	0
CK-10	2	1	1	3	0	0	1
NE	0	2	0	0	0	2	3
AQP-9	1	2	2	2	0	2	3
Fibrinogen	2	2	2	2	0	2	3
CK-9	1	1	3	0	3	2	2
CK-2	1	2	3	2	3	2	2
CK-1	3	2	2	0	2	2	3
IgG	3	3	2	2	2	3	3
SNA	2	3	2	3	3	3	2
PAD4	3	3	2	3	2	3	3
GalNAc	3	3	3	3	3	3	3

[1] The intensity of various IF stainings was visually estimated for each MPC-D using a 0-3 point scale (no to high antigen abundance). The core of the tissue (upper part of the table) was evaluated separately from the surface (lower part of the table). All antigens were sorted according to their abundance, from lowest to highest.
